# A systematic review and meta-analysis of complications of artificial urinary sphincters in female patients with urinary incontinence due to internal sphincter insufficiency

**DOI:** 10.1186/s12894-023-01274-x

**Published:** 2023-05-20

**Authors:** Pourya Shokri, Ladan Kharaz, Niki Talebian, Nasrin Borumandnia, Seyed Amir Mohsen Ziaee, Nasser Shakhssalim

**Affiliations:** 1 Shahid Beheshti University of Medical Sciences, Tehran, Iran; 2 Urology and Nephrology Research Center, Shahid Beheshti University of Medical Sciences, Tehran, Iran; 3 Labbafinejad Medical Center, Pasdaran, Tehran, Iran

**Keywords:** Artificial urinary sphincter, Internal sphincter, Stress incontinence, Complete incontinence, Complication

## Abstract

**Background:**

Urinary incontinence (UI) is a common worldwide rising health issue among women with a prevalence of 5 to 70%. Stress urinary incontinence (SUI) is the most common subtype of UI. There are different treatments for UI, including AUS (artificial urinary sphincter) implantation, as one of the surgical options for treating SUI. The aim of this study was to determine the complication rate of AUS, exclusively in female patients with SUI, which resulted from ISD (intrinsic sphincter deficiency). We also compared the complication rate between minimally invasive (laparoscopic or robotic surgery) and open approaches.

**Methods:**

Scopus, PubMed, Web of Science, Embase, and Google Scholar were searched for studies regarding complications in AUS implantation surgery, from the beginning of the project to March 2022. After screening and reviewing of full text, the general characteristics of the study and study population including follow-up time, type of surgery, and the number of complications that occurred such as necrosis, atrophy, erosion, infection, mechanical failure, revision, and leak, were extracted.

**Results:**

We found that atrophy occurred in 1 of 188 (0.53%) patients treated with minimally invasive surgery and in 1 of 669 (0.15%) patients treated with open surgery. None of the 17 included studies reported the occurrence of necrosis in the patients under study. Erosion occurred in 9 of 188 (4.78%) patients treated with minimally invasive surgery and in 41 of 669 (6.12%) patients treated with open surgery. Infection occurred in 12 of 188 (6.38%) patients treated with minimally invasive surgery and in 22 of 669 (3.2%) patients treated with open surgery. The mechanical failure occurred in 1 of 188 (0.53%) patients treated with minimally invasive surgery and in 55 of 669 (8.22%) patients treated with open surgery. Reconstructive surgery occurred in 7 of 188 (3.72%) patients treated with minimally invasive surgery and in 95 of 669 (14.2%) patients treated with open surgery. Leaks occurred in 4 of 188 (2.12%) patients treated with minimally invasive surgery and in 6 of 669 (0.89%) patients treated with open surgery. The type of surgery was associated with a statistically significant increase in mechanical failure (p-value = 0.067) and infection (p-value = 0.021), and reconstructive surgery (p-value = 0.049). Out of the 857 participats in the study,469 were studied for less than five years and 388 were studied for more than five years.21 of 469 (4.4%) (p-value = 0.08) patients and 81 of 388 (20.8%) (p-value = 0.001) patients required reconstructive surgery. Erosion occurred in 23 of 469 (4.9%) (p-value = 0.01)patients with following time less than five years and in 27 of 388 (6.9%) (p-value = 0.001) patients with following time more than five years.

**Conclusion:**

The use of artificial urinary sphincters in the treatment of UI causes complications such as atrophy, erosion, and infection; the amount of which is influenced by the surgical method and the duration of using the artificial urinary sphincter. It seems that the use of new surgical methods, such as laparoscopic surgery, is useful in reducing the incidence of complications.

## Introduction

Urinary incontinence (UI) is a worldwide rising health issue among women, it affects the quality of life and self-esteem and even is associated with depression and stress [1–3]. Based on a review study, the prevalence of UI varies from 5 to 70% [4]. The most common subtype of UI is stress urinary incontinence (SUI) [3]. SUI prevalence varies by age and ethnicity and rises by age, and a recent study indicated that it has a prevalence of 37.1% in old adults [5]. Two main known mechanisms that are causing SUI are urethral hypermobility and intrinsic sphincter deficiency (ISD) [6].

There are options to treat female patients with SUI caused by ISD, including bulking agents, different types of slings, balloons, and AUS [7, 8] but only AUS can imitate the physiological function of the sphincter and it is the gold-standard treatment of UI in men and one of efective treatments for women, in situations when non-invasive conservative and medical therapies fail [9–11].

The concept of AUS was suggested by Foley for the first time in 1947, and after 25 years, the first AUS made by the American Medical Systems (AMS) 721 (Minnetonka, MN, USA) was used in practice. The newest AUS AMS 800 is released after years of modification and development [12].

Based on the guidelines of 2020 of the European Association of Urology (EAU), the first steps of SUI management are treating the underlying disease, lifestyle modification, and behavioral and physical therapies, including pelvic floor muscle training (PFMT), and pharmacological therapy. Surgical options are recommended for treating patients who failed to respond to conservative or medical therapies. First-line surgeries that are recommended for women with uncomplicated SUI are inserting a mid-urethral sling, colposuspension, or autologous fascial sling.

No strong evidence exists to support assigning AUS to women [13] and also there are not sufficient patient populations and follow-up time to conclude about laparoscopically implanted AUS [14].

As a result, AMS-800 AUS may have favorable effects on women with SUI, caused by ISD and more studies must be done about the complications of this treatment, especially for new surgical techniques including laparoscopic and robotic approaches [10].

The aim of this study was to determine the complication rate of AUS, exclusively in female patients with SUI due to ISD. We also compared the complication rate between minimally invasive and open approaches.

## Materials and methods

### Search strategy

We conducted this study to investigate the prevalence of complications in AUS implantation surgery for women with SUI due to ISD, and in accordance with the PRISMA guidelines [15]. We searched Scopus, PubMed, Web of Science, Embase, and Google Scholar from the beginning of the project to March 2022 for studies regarding complications in AUS implantation surgery. Keywords were applied to search for proper studies, including ‘incontinency’, ‘internal sphincter deficiency’, ‘complication’, ‘atrophy’, ‘erosion’, ‘necrosis’, ‘revision’, ‘mechanical failure’, ‘infection’, ‘urethral sphincter’, ‘urinary sphincter’, ‘urinary incontinence’, ‘AUS’, ‘artificial bladder sphincter’, ‘female urethral sphincter’, ‘artificial sphincter’, ‘female artificial sphincter’, and ‘AMS sphincter 800’. In the initial screening, 3115 related studies were included and then 308 articles were selected based on the following criteria, including (1) AUS implantation for women with UI due to ISD, (2) original studies, and (3) studies published in English. The exclusion criteria were animal studies and studies presented in meetings /congresses that were not peer-reviewed. All these steps were done by two independent researchers with the consultation and revision of a third researcher (Table 1).

**Table 1:** Description of cohorts of patients included in the current evaluation

Study	Design of study	Quality	Complications							Mean group age	type of sphincter	Cuff size	Type of surgery	Sample size	Follow-up time (month)
			Atrophy	Necrosis	Erosion	Infection	M.F	Revision	Leak						
Gondrantellier [17]	p. cohort	6	0	0	0	0	0	1	1	64 (58–76)	AMS-800	8 cm	M.I	8	12(6–27) SH.T
Fournierg [18]	p. cohort	5	0	0	0	0	0	0	1	65 (50–75)	AMS 800	8 ± 0.8 cm	M.I	6	14.3 ± 9 (3–21), SH.T
Ngninkeu [19]	R. cohort	4	0	0	0	0	0	0	1	68.5 (50–79)	AMS 800		M.I	4	6 (3–13), SH.T
Peyronnet [20]	R. cohort	6	1	0	1	4	1	3	0	70.5 (28–86)	AMS-800	7 cm (5–9)	M.I	49	18.5 (12–64), SH.T
Phé [21]	R. cohort	5	0	0	1	6	3	12	0	56.5 (50–64.7)	AMS-800	5–10 cm	Open	34	204, L.T (144–228)
Roupreˆt [14]	R. cohort	5	0	0	0	0	0	0	1	56.7 (33–78)	AMS 800	6–7 cm	M.I	12	12.1 ± 8 (5.2–27), SH.T
Tricard [22]	R. cohort	5	1	0	5	0	0	3	0	58 (17–82)	AMS 800		Open	63	168 ± 72 L.T
Vayleux [23]	R. cohort	5	0	0	10	3	0	33	0	62.8 (25–85)	AMS 800	6.5–7.5 cm	Open	215	72.3 ± 67 L.T
George [24]	R. cohort	5	0	0	0	0	0	4	2	61 (19–79)	AS792 + AS-800	6.5 cm (5.5–8)	Open	25	31.2 (12–106.8), SH.T
Biardeau [25]	R. cohort	5	0	0	3	1	0	2	0	66 (63–72)	AUS	6.5 cm (5.5–7.25)	M.I	11	17.6(10.8–25.95), SH.T
Bracchitta [26]	R. cohort	6	0	0	5	4	0	0	0	66.9	AMS 800	4.5–9 cm	M.I	74	54, SH.T
Broudeur [27]	R. cohort	6	0	0	0	3	0	1	0	66 (61–74)	AUS	7.5 cm (7.5–8.0)	M.I	24	26 (22–36) SH.T
Chung [28]	R. cohort	6	0	0	6	2	18	20	0	51 (17–78)	AMS 800		Open	47	162 (36–300) L.T
Chung [29]	R. cohort	6	0	0	5	5	12	13	0	54.5 (29–77)	AUS	6 cm	Open	29	106.1, L.T (24–216)
Costa [30]	R. cohort	7	0	0	12	1	6	0	2	59.2 ± 11	AMS 800		Open	179	46.8 ± 28,8 SH.T
Denormandie[31]	R. cohort	5	0	0	2	5	9	9	0	77 (75–79)	AMS 800	5–9 cm	Open	45	36 (16–96), SH.T
Diokno [32]	R. cohort	6	0	0	0	0	7	1	2	55 (32–82)	AMS 800 + other types		Open	32	30 (6–72), SH.T

Included patients for pooled analysis were those with urinary incontinence due to internal sphincter disorder treated by artificial urinary sphincter through minimal invasive or open surgery

*P. cohort* prospective cohort; *M.F* mechanical failure; *R. cohort* retrospective cohort; *M.I* minimal invasive; *SH.T* short term; *L.T* long term

### Data extraction and quality assessment

Two independent reviewers read the abstract of articles that matched the aforementioned criteria and then reviewed the full text of selected articles in detail. Then, the general characteristics of the study (title, first author, year of study, year of publication, place of study, design, quality, and follow-up time), characteristics of the study population (sample size, number of females, type, and size of the sphincter that is used, mean age, the standard deviation of age, comorbidities, body mass index (BMI)) and number of complications that occurred (necrosis, atrophy, erosion, infection, mechanical failure, revision, and leak) were extracted. Evaluation of included studies was done by using the Newcastle–Ottawa quality assessment for cohort studies [16]. Studies were evaluated in terms of criteria such as: 1. Selection (Representativeness of the exposed cohort,Selection of the non exposed cohort, Ascertainment of exposure, Demonstration that outcome of interest was not present at start of study) 2. Comparability (Comparability of cohorts on the basis of the design or analysis) 3.Outcome (Assessment of outcome, Was followup long enough for outcomes to occur, Adequacy of follow up of cohorts). Then,the studies were divided into three groups(good, fair, poor) based on the quality score (Table 2).

**Table 2:** Newcastle–Ottawa quality assessment form for cohort studies

Study identification	DOP	Selection				Comparability	Outcome			9. Overall score
		1. Representativness of the exposed cohort	2. Selection of the non-exposed cohort	3. Ascertainment of exposure	4. Demonstration that outcome of interest was not present at start of study	5. Comprability of cohort on the basis of the design or analysis	6. Assessment of outcome	7. Was follow-up long enough for outcomes to occur	8. Adequacy of follow up of cohorts	
Gondran-Tellier [1]	2019	a	c	a	a	a	c	a	a	6
Fournierg [2]	2014	c	c	a	a	a	c	a	a	5
Ngninkcj [3]	2005	d	c	a	a	c	d	a	a	4
Peyronnet [4]	2018	a	c	a	a	c	a	a	a	6
Phé [5]	2014	a	c	a	a	c	d	a	a	5
Rouprêt [6]	2009	a	c	a	a	c	d	a	a	5
Tricard [7]	2019	a	c	a	a	c	d	a	a	5
Vayleux [8]	2011	a	c	a	a	c	d	a	a	5
George [9]	1992	a	c	a	a	c	d	a	a	5
Biardeau [10]	2015	c	c	a	a	a	c	a	a	5
Bracchitta [11]	2019	a	c	a	a	a	c	a	a	6
Broudeur [12]	2021	a	c	a	a	a	c	a	a	6
Chung [13]	2010	a	c	a	a	a	d	a	a	6
Chung [14]	2010	a	c	a	a	a	c	a	a	6
Costa [15]	2001	a	c	a	a	a	a	a	a	7
Denormandie [16]	2021	b	c	a	a	a	c	a	a	5
Diokno []	1987	a	c	a	a	a	c	a	a	6

### Statistical analysis

Meta-analysis was performed to estimate the prevalence of each complication. Also, the meta-analysis was stratified according to surgery type and follow-up time. If a report included an explicit prevalence of 0%, we increased the prevalence slightly, to 0.2%, to calculate the standard error (SE) and provide an approximate 95% CI. When the heterogeneity index of I^2^ was lower than 50%, the pooled prevalence was calculated, using a fixed-effects model. When the heterogeneity index of I^2 was higher than 50%, a random-effects model was used. The studies are weighted using the inverse of the variance. The results are presented using Forrest graphs. The heterogeneity of the prevalence between studies was estimated for each surgery type and follow-up time category. Meta-regression was used to determine whether a significant difference was present between surgery groups. The statistical software package STATA 14.0 (StataCorp LLC., College Station, TX) was used to perform the analysis. In this study, p-value less than 0.05 was considered significant.

## Results

### Study identification and selection

**Fig. 1:**
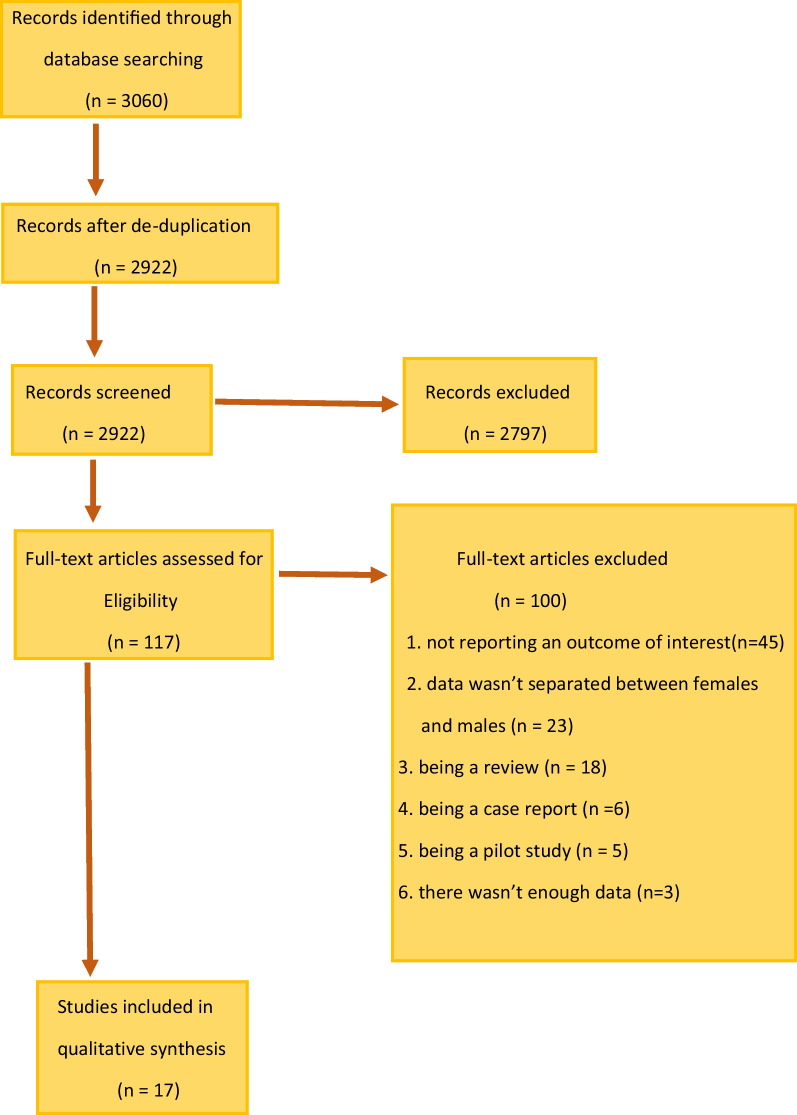
PRISMA study selection flow chart

For data extraction, we searched through Scopus, PubMed, Web of Science, Embase, and Google Scholar databases and used PRISMA 2020 template for the study design. The initial search identified 3060 articles (Fig. 1). After de-duplication, 2922 articles were screened for further analysis, out of which 2797 were excluded upon the initial screening, leaving 117 studies for the full review. Of the 117 studies, 45 were excluded due to not reporting an outcome of interest (n = 45), or the data were not separated between women and men (n = 23), or because of being a review (n = 18), a case report (n = 6), a pilot study (n = 5), or there was not enough data (n = 3).

### Description of included studies

Seventeen studies were included, comprising 857 female patients with SUI caused by ISD, using AUS. Of these, 736 were using AMS 800 (American Medical Systems, Inc., Minnetonka, Minnesota) AUS (n = 12), and 121 were using other types of AUS (n = 5). About 188 had undergone minimally invasive surgery (n = 8) and 669 had undergone open surgery (n = 9). The mean age ranged from 56.7 to 70.5 years for patients treated through minimally invasive surgery and 51 to 77 years for patients treated through open surgery. The cuff size ranged from 4.5 to 9 cm for patients undergoing minimally invasive surgery and 5 to 10 cm for patients undergoing open surgery (Table 1).

We divided the follow-up studies into (1) short-term follow-up (mean follow-up < 5 years), including 469 women (n = 12), of which 188 had undergone minimally invasive procedures (n = 8) and 281 of them had undergone open procedures (n = 4), and (2) long term follow-up (mean follow-up > 5 years) includes 388 women (n = 5) that all of them had undergone open procedure (Table 1).

In this study, we compared the complications of AUS among the population undergone minimally invasive and open surgeries. Complications comprised some necrosis, atrophy, erosion, infection, mechanical failure, revision needing, and leak (sphincter insufficiency) (Table 1).

### Quality assessment of included studies

The results of the quality assessment are summarized in Table 2. Using the criteria that a study with 2 stars in the selection domain AND 1 or 2 stars in the comparability domain AND 2 or 3 stars in the outcome/exposure domain of the Newcastle–Ottawa quality assessment for cohort studies, was considered to be fair quality. Eight studies had good qualities, eight studies had fair qualities and one study had poor quality(Table 2). Most of the studies were designed as retrospective cohort studies and hence were subject to the lacking of data that could be affecting the results. Lacking data in studies included the selection of the non-exposed variables in cohorts, lack of result assessment description, lack of data related to cuff size and type of sphincter in some of the studies, and in case competing interests and source of funding, were not reported.

### Atrophy

Out of 857 patients under study, 2 cases of atrophy were reported (0.05 [95% CI, 0–0.19], *P* = 1.00, I2 = 0%). Atrophy occurred in 1 of 188 (0.53%) patients treated with minimally invasive surgery and in 1 of 669 (0.15%) patients treated with open surgery, in the 17 included studies (Table 1). The type of surgery was not associated with a statistically significant increase in atrophy number (p value = 0.713). The pooled effect size was 0.14% (0%, 0.69%) in patients treated with minimally invasive surgery and 0.04% (0%, 0.19%) in patients treated with open surgery (Table 3). In addition, the pooled effect size was 0.06% (0.0%, 0.27%) among the population with a follow-up of < 5 years and 0.04% (0%, 0.23%) among the population with a follow-up of > 5 years (Table 4).

**Table 3:** Meta-analysis results based on open procedure and laparoscopic

Outcome	Subgroup	Pooled effect size (CI)	Test ES = 0		I^2^* (%)
			Z score	p-value	
Atrophy	laparoscopic	.14% (0%, .69%)	.52	.60	0
	Radical	.04% (0%, .19%)	.51	.61	0
Erosion	laparoscopic	1.65% (0%, 3.43%)	1.78	.08	9.57
	Radical	4.36% (1.79%, 6.92%)	3.33	< .001	54.6
Infection	laparoscopic	3.96% (1.22%, 6.71%)	2.83	< .001	0
	Radical	1.21% (0%, 2.49%)	1.86	.06	61.63
Mechanical failure	laparoscopic	.28% (0%, 1.04%)	.73	.46	0
	Radical	4.51% (1.96%, 7.06%)	3.47	< .001	89.82
Revision needing	laparoscopic	.98% (0%, 2.69%)	1.12	.26	7.56
	Radical	17.73% (9.55%, 25.92%)	4.25	< .001	94.04
Leak (sphincter insufficiency)	laparoscopic	.21% (0%, .90%)	.60	.55	0
	Radical	.11% (0%, .37%)	.81	.42	0

**Table 4:** Meta-analysis results based on the follow up period (short term [less than 5 years] and long term [more than 5 years])

Outcome	Subgroup (years)	Pooled effect size (CI)	Test ES = 0		I^2^* (%)
			Z score	p-value	
Atrophy	< 5	.06% (.0%, .27%)	.51	.61	0
	> 5	.04% (0%, .23%)	.39	.70	0
Erosion	< 5	1.52% (.43%, 2.61%)	2.74	.01	0
	> 5	5.55% (3.29%, 7.81%)	4.82	< .001	0
Infection	< 5	.90% (.06%, 1.74%)	2.13	.03	31
	> 5	2.58z (0%, 5.34%)	1.84	.07	74.45
Mechanical failure	< 5	1.78% (.05%, 3.50%)	2.02	.04	56.22
	> 5	4.03% (.77%, 7.28%)	2.42	.02	92.42
Revision needing	< 5	1.29% (0%, 2.73%)	1.75	.08	55.53
	> 5	26.53% (13.15%, 39.91%)	3.89	< .001	91.24
Leak (sphincter insufficiency)	< 5	.39% (0%, .96%)	1.33	.18	0
	> 5	.06% (0%, .33%)	.44	.66	0

### Necrosis

None of the 17 included studies reported the occurrence of necrosis in the patients under study (Table 1).

### Erosion

**Fig. 2:**
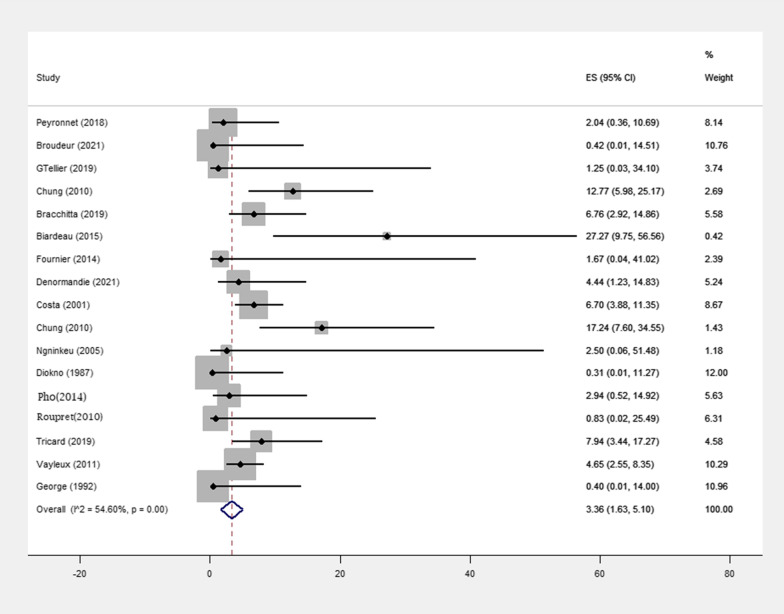
Meta-analysis for number of erosion in female patients with urinary incontinence due to intrinsic sphincter insufficiency

A total of 17 studies reported 50 erogenous events number (p-value = < 0.001) (Fig. 2). Erosion occurred in 9 of 188 (4.78%) patients treated with minimally invasive surgery and in 41 of 669 (6.12%) patients treated with open surgery (Table 1). The pooled effect size was 1.65% (0%, 3.43%) in patients treated with minimally invasive surgery and 4.36% (1.79%, 6.92%) in patients treated with open surgery (Table 3). Also, the pooled effect size was 1.52% (0.43%, 2.61%) among the population with a follow-up < 5 years and 5.55% (3.29%, 7.81%) among the population with a follow-up > 5 years (Table 4).

### Infection

**Fig. 3:**
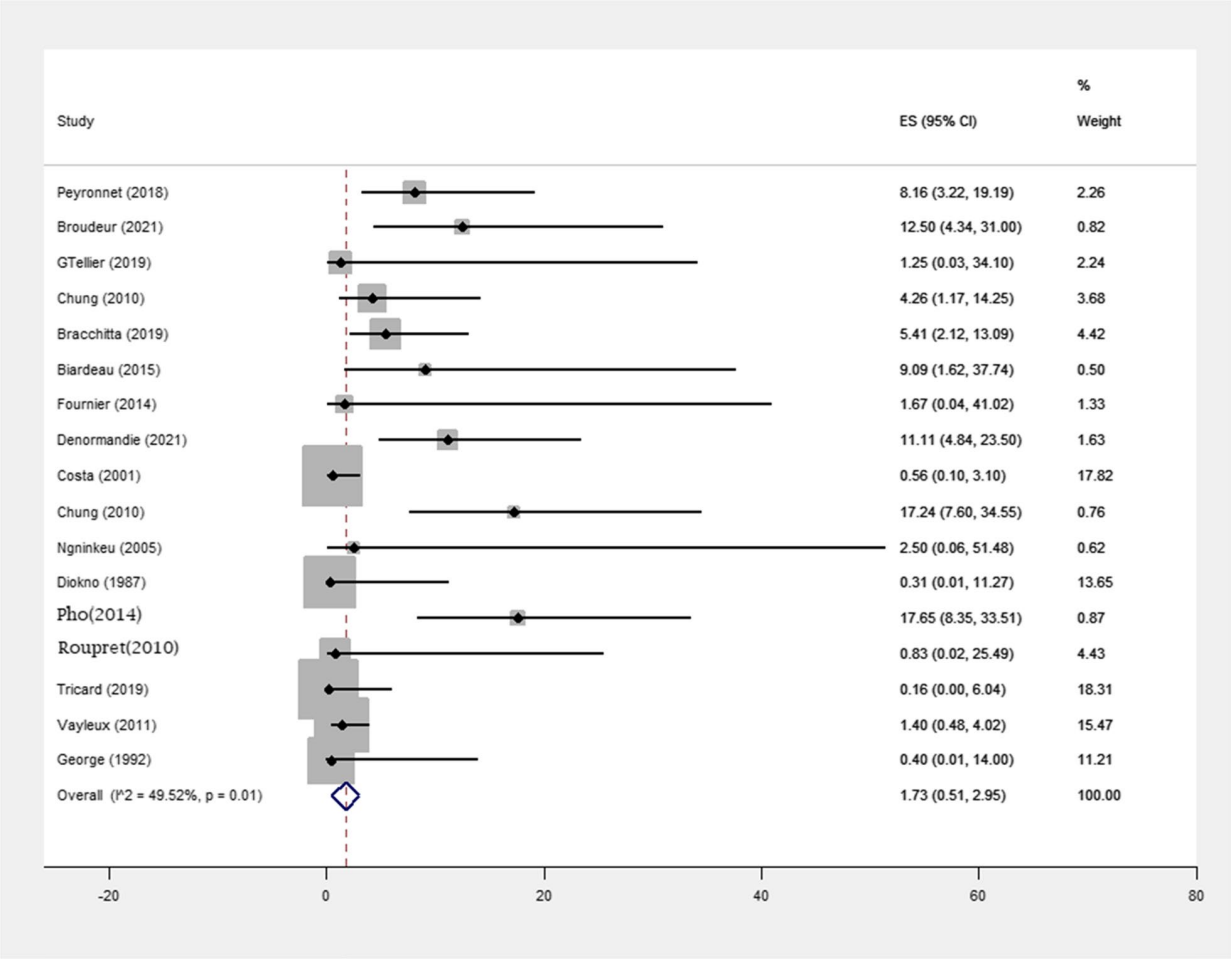
Meta-analysis for number of infection in female patients with urinary incontinence due to intrinsic sphincter insufficiency

Infection occurred in 34 of 857 (3.9%) patients, among them 12 out of 188 (6.38%) patients were treated with minimally invasive surgery and 22 out of 669 (3.2%) patients treated with open surgery, in the 17 included studies (p-value = 0.01) (Table 1). The mean follow-up period was associated with a statistically significant increase in infection numbers (p value = 0.21) (Fig. 3). The pooled effect size was 3.96% (1.22%, 6.71%) in patients treated with minimally invasive surgery and 1.21% (0%, 2.49%) in patients treated with open surgery (Table 3). In open surgery, the chance of infection is lower than in minimally invasive surgery(p-value = 0.021)(Table 5). Also, the pooled effect size was 0.90% (0.06%, 1.74%) among the population with a follow-up of < 5 years and 2.58% (0%, 5.34%) among the population with a follow-up of > 5 years (Table 4).

**Table 5:** Meta-regression analysis to compare open procedure versus laparoscopic (reference)

Outcome	Coefficient	Standard error	p-value
Atrophy	− 0.10	0.28	.713
Erosion	1.91	2.01	.356
Infection	− 3.31	1.43	.021
Mechanical failure	10.37	5.25	.067
Revision needing	13.23	6.19	.049
Leak (sphincter insufficiency)	− 0.10	0.37	.788

### Mechanical failure

A mechanical failure occurred in 56 of 857 (6.5%) patients, including 1 of 188 (0.53%) patients treated with minimally invasive surgery and 55 of 669 (8.22%) patients treated with open surgery, in the 17 included studies (p value = 0.01) (Table 1). The mean follow-up period was associated with a statistically significant increase in mechanical failure numbers. The pooled effect size was 0.28% (0%, 1.04%) in patients treated with minimally invasive surgery and 4.51% (1.96%, 7.06%) in patients treated with open surgery (Table 3). Also, the pooled effect size was 1.78% (0.05%, 3.50%) among the population with a follow-up of < 5 years and 4.03% (0.77%, 7.28%) among the population with a follow-up of > 5 years (Table 4).

### Reconstructive surgery

**Fig. 4:**
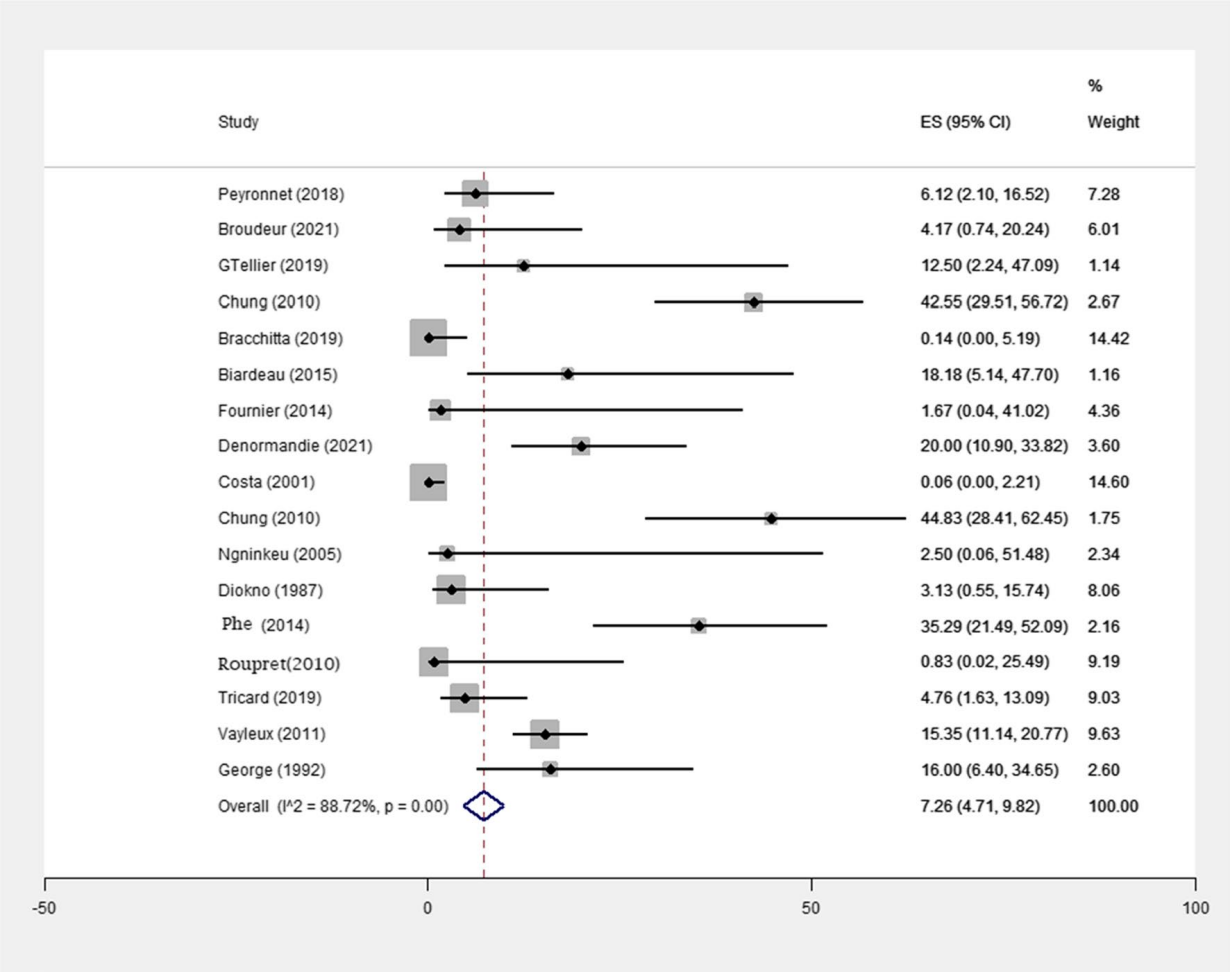
Meta-analysis for number of revision needing in female patients with urinary incontinence due to intrinsic sphincter insufficiency

Reconstructive surgery occurred in 102 of 857 (11.9%) patients, including 7 of 188 (3.72%) patients treated with minimally invasive surgery and in 95 of 669 (14.2%) patients treated with open surgery, in the 17 included studies (p value = 0 < 001) (Table 1). The mean follow-up period was associated with a statistically significant increase in reconstructive surgery numbers (Fig. 4). The pooled effect size was 0.98% (0%, 2.69%) in patients treated with minimally invasive surgery and 17.73% (9.55%, 25.92%) in patients treated with open surgery (Table 3).In Comparing open surgery methods with minimally invasive surgery methods, the probability of reconstructive surgery in patients treated with minimally invasive surgery methods is lower than open surgery methods(p value = 0.049)(Table 5). In addition, the pooled effect size was 1.29% (0%, 2.73%) among the population with a follow-up of < 5 years and 26.53% (13.15%, 39.91%) among the population with a follow-up of > 5 years (Table 4).

### Leak

Leaks occurred in 10 of 857 (1.1%) patients, including 4 of 188 (2.12%) patients treated with laparoscopic surgery and in 6 of 669 (0.89%) patients treated with open surgery, in the 17 included studies (p value = 0.33) (Table 1). The type of surgery was associated with a statistically significant increase in leak number (p value = 0.788). The pooled effect size was 0.21% (0%, 0.90%) in patients treated with minimally invasive surgery and 0.11% (0%, 0.37%) in patients treated with open surgery (Table 3). Also, the pooled effect size was 0.39% (0%, 0.96%) among the population with a follow-up of < 5 years and 0.06% (0%, 0.33%) among the population with a follow-up of > 5 years (Table 4).

## Discussion

There are various treatment methods to treat UI, especially in women, like a sling, and AUS, which provide acceptable results. Retropubic and trans-obturator mid-urethral slings are common methods for the treatment of women with SUI but this treatment method can cause complications and voiding lower urinary tract symptoms, and in some cases with a severe form of UI cannot provide sufficient UI. One of the effective treatments for women with UI is the usage of artificial urinary sphincters, which is the gold-standard treatment for males [33]. The use of sphincters is always associated with the possibility of complications, which can be related to the surgical method of sphincter insertion or radiation, catheterization, and diabetes mellitus [33, 34].

According to the results of this study, the probability of erosions and infection, which are important and common side effects of using artificial urinary sphincters, are generally 3.36% and 1.73%, respectively [35]. The probability of necrosis is higher in patients who have a history of radiotherapy [36] but in general, necrosis is a rare complication and there was no report of necrosis in our study. The duration of using an artificial urinary sphincter has an important effect on the probability of some side effects, so the probability of reconstructive surgery in less than 5 years was 1.29% and in the long term it was 26.53%. Also, the probability of erosion in less than 5 years, was 1.52% and in the long term, the probability of its occurrence reached 5.55%.

Today, less invasive surgical methods are used for implantation, which has changed the probability of side effects from using artificial urinary sphincters [37]. The probability of reconstructive surgery in patients who have a sphincter implanted by laparoscopic or open surgery was 17.73% in open surgery and 0.98% in laparoscopy. The probability of infection in open surgery was 1.21% and in the laparoscopic method was 3.96%, and the probability of atrophy in open surgery was 0.04% and in laparoscopy was 0.14%.

Another problem that can occur is the occurrence of a sphincter disorder, which can be due to damage to the cuff or tube or pump or leakage of the sphincter liquid source, depending on the type of sphincter used, the duration of using the sphincter, and even the method of its operation. According to the statistics of this study, the probability of occurrence of mechanical failure in the open surgery method was 4.51% and in laparoscopy was 0.28%, also the probability of occurrence in less than 5 years was 1.78%, and in the long-term was 4.03% [37].

In general, according to the information obtained from other studies, the use of artificial urinary sphincters compared to other methods such as sling or gel injection can cause many unwanted side effects. In this study, we presented the statistics of 17 main studies on female patients who had UI due to dysfunction of the internal urethral sphincter and used artificial urinary sphincters for treatment.

Most of these studies are retrospective studies that have followed patients in different periods, in terms of the occurrence of complications, and the final results show the acceptable performance of artificial urinary sphincters in these patients, so the use of artificial urinary sphincters is an alternative. The method is recommended in patients when other treatment methods have not been effective.

This study was conducted with the aim of evaluating the complications caused by the use of artificial urinary sphincters in women with internal urethral sphincter defects. Nevertheless, there are some limitations in this study that should be considered when interpreting the results. Firstly, we only included studies published in English language. Secondly, many studies which conducted in this field did not accurately and statistically state the cause of urinary incontinence in patients. Therefore, we could not use these studies in our statistical analysis. Thirdly, the protocol of this study was not registered in PROSPERO, although the necessary ethical approvals were obtained from the relevant university committee.

## Conclusion

Finally, the use of artificial urinary sphincters can be associated with complications such as necrosis, atrophy, erosion, infection, and the need for re-surgery, and the probability of these complications is affected by the duration of sphincter use and the method of implantation. So that the possibility of complications increases with time. New surgical methods such as laparoscopic surgery have reduced the possibility of some complications, including erosion but due to the limited statistical population of laparoscopic data, further studies are needed to investigate these surgical methods.

## Data Availability

The data that support the findings of this study are available upon reasonable request from the corresponding author.

## References

[1] Lee H-y, Rhee Y, Choi KS (2021). Urinary incontinence and the association with depression, stress, and self-esteem in older Korean Women. Sci Rep.

[2] Pandey D, Maturi C, Dhakar BPS, Jain G, Kyalakond K (2019). Interventions and quality of life in stress urinary incontinence. Gynecol Minim Invasive Ther.

[3] Abufaraj M, Xu T, Cao C, Siyam A, Isleem U, Massad A (2021). Prevalence and trends in urinary incontinence among women in the United States, 2005–2018. Am J Obstet Gynecol.

[4] . Milsom I, Altman D, Cartwright R, Lapitan M, Nelson R, Sillén U, et al. Epidemiology of urinary incontinence (UI) and other lower urinary tract symptoms (LUTS), pelvic organ prolapse (POP) and anal incontinence (AI). Incontinence: 5th International Consultation on Incontinence, Paris, February 2012: ICUD-EAU; 2013. p. 15–107.

[5] Mckellar K, Abraham N (2019). Prevalence, risk factors, and treatment for women with stress urinary incontinence in a racially and ethnically diverse population. Neurourol Urodyn.

[6] Bennington J, Williams JK, Andersson K-E (2019). New concepts in regenerative medicine approaches to the treatment of female stress urinary incontinence. Curr Opin Urol.

[7] Hillary CJ, Osman N, Chapple C (2015). Considerations in the modern management of stress urinary incontinence resulting from intrinsic sphincter deficiency. World J Urol.

[8] Osman NI, Marzi VL, Cornu JN, Drake MJ (2016). Evaluation and classification of stress urinary incontinence: current concepts and future directions. Eur Urol Focus.

[9] Cour F, Le Normand L, Lapray J, Hermieu J, Peyrat L, Yiou R (2015). Intrinsic sphincter deficiency and female urinary incontinence. Prog Urol J L'assoc Fr D'urol Soc Fr D'urol.

[10] Peyronnet B, O'Connor E, Khavari R, Capon G, Manunta A, Allue M (2019). AMS-800 artificial urinary sphincter in female patients with stress urinary incontinence: a systematic review. Neurourol Urodyn.

[11] Marziale L, Lucarini G, Mazzocchi T, Gruppioni E, Castellano S, Davalli A (2018). Artificial sphincters to manage urinary incontinence: a review. Artif Organs.

[12] Ratan HL, Summerton DJ, Wilson SK, Terry TR (2006). Development and current status of the AMS 800 artificial urinary sphincter. EAU-EBU update series.

[13] Chung E, Cartmill RA (2010). 25-year experience in the outcome of artificial urinary sphincter in the treatment of female urinary incontinence. BJU Int.

[14] Rouprêt M, Misraï V, Vaessen C, Cardot V, Cour F, Richard F (2010). Laparoscopic approach for artificial urinary sphincter implantation in women with intrinsic sphincter deficiency incontinence: a single-centre preliminary experience. Eur Urol.

[15] Liberati A, Altman DG, Tetzlaff J, Mulrow C, Gøtzsche PC, Ioannidis JP (2009). The PRISMA statement for reporting systematic reviews and meta-analyses of studies that evaluate health care interventions: explanation and elaboration. J Clin Epidemiol.

[16] Wells G, Shea B, O’Connell D, Peterson J, Welch V, Losos M (2014). Newcastle-Ottawa quality assessment scale cohort studies.

[17] Gondran-Tellier B, Boissier R, Baboudjian M, Rouy M, Gaillet S, Lechevallier E (2019). Robot-assisted implantation of an artificial urinary sphincter, the AMS-800, via a posterior approach to the bladder neck in women with intrinsic sphincter deficiency. BJU Int.

[18] Fournier G, Callerot P, Thoulouzan M, Valeri A, Perrouin-Verbe MA (2014). Robotic-assisted laparoscopic implantation of artificial urinary sphincter in women with intrinsic sphincter deficiency incontinence: initial results. Urology.

[19] . Ngninkeu BN, van Heugen G, di Gregorio M, Debie B, Evans A. Laparoscopic artificial urinary sphincter in women for type III incontinence: preliminary results. Eur Urol. 2005;47(6):793–7; discussion 7. 10.1016/j.eururo.2005.01.010 15925075

[20] Peyronnet B, Capon G, Belas O, Manunta A, Allenet C, Hascoet J (2019). Robot-assisted AMS-800 artificial urinary sphincter bladder neck implantation in female patients with stress urinary incontinence. Eur Urol.

[21] Phé V, Benadiba S, Rouprêt M, Granger B, Richard F, Chartier-Kastler E (2014). Long-term functional outcomes after artificial urinary sphincter implantation in women with stress urinary incontinence. BJU Int.

[22] Tricard T, Jochum F, Bergerat S, Munier P, Schroeder A, Saussine C (2019). Outcomes of open artificial urinary sphincter in women with stress urinary incontinence: long-term follow up. Ther Adv Urol.

[23] Vayleux B, Rigaud J, Luyckx F, Karam G, Glémain P, Bouchot O (2011). Female urinary incontinence and artificial urinary sphincter: study of efficacy and risk factors for failure and complications. Eur Urol.

[24] Webster GD, Perez LM, Khoury JM, Timmons SL (1992). Management of type III stress urinary incontinence using artificial urinary sphincter. Urology.

[25] Biardeau X, Rizk J, Marcelli F, Flamand V (2015). Robot-assisted laparoscopic approach for artificial urinary sphincter implantation in 11 women with urinary stress incontinence: surgical technique and initial experience. Eur Urol.

[26] Bracchitta D, Costa P, Borojeni S, Ménard J, Bryckaert PE, Mandron É (2019). Laparoscopic artificial urinary sphincter implantation in women with stress urinary incontinence: update on 13 years' experience in a single centre. BJU Int.

[27] Broudeur L, Loubersac T, Le Normand L, Karam G, Branchereau J, Rigaud J (2021). New technique of robot-assisted laparoscopic artificial urinary sphincter implantation in female by a posterior approach with intraoperative cystoscopic monitoring. World J Urol.

[28] Chung E, Cartmill RA (2010). 25-year experience in the outcome of artificial urinary sphincter in the treatment of female urinary incontinence. BJU Int.

[29] Chung E, Navaratnam A, Cartmill RA (2011). Can artificial urinary sphincter be an effective salvage option in women following failed anti-incontinence surgery?. Int Urogynecol J.

[30] Costa P, Mottet N, Rabut B, Thuret R, Ben Naoum K, Wagner L (2001). The use of an artificial urinary sphincter in women with type III incontinence and a negative Marshall test. J Urol.

[31] Denormandie A, Chartier-Kastler E, Haddad R, Robain G, Guillot-Tantay C, Phé V (2021). Long-term functional outcomes of artificial urinary sphincter (AMS 800™) implantation in women aged over 75 years and suffering from stress urinary incontinence caused by intrinsic sphincter deficiency. World J Urol.

[32] Diokno AC, Hollander JB, Alderson TP (1987). Artificial urinary sphincter for recurrent female urinary incontinence: indications and results. J Urol.

[33] Khouri RK, Ortiz NM, Dropkin BM, Joice GA, Baumgarten AS, Morey AF (2021). Artificial urinary sphincter complications: risk factors, workup, and clinical approach. Curr Urol Rep.

[34] Schroeder A, Munier P, Saussine C, Tricard T (2021). Outcomes of laparoscopic artificial urinary sphincter in women with stress urinary incontinence: mid-term evaluation. World J Urol.

[35] Reus CR, Phé V, Dechartres A, Grilo NR, Chartier-Kastler EJ, Mozer PC (2020). Performance and safety of the artificial urinary sphincter (AMS 800) for non-neurogenic women with urinary incontinence secondary to intrinsic sphincter deficiency: a systematic review. Eur Urol Focus.

[36] Doiron RC, Witten J, Rourke KF (2021). The scope, presentation, and management of genitourinary complications in patients presenting with high-grade urethral complications after radiotherapy for prostate cancer. Can Urol Assoc J.

[37] Peyronnet B, O'Connor E, Khavari R, Capon G, Manunta A, Allue M (2019). AMS-800 Artificial urinary sphincter in female patients with stress urinary incontinence: a systematic review. Neurourol Urodyn.

